# Data quality assessment of the Enhanced Gonococcal Antimicrobial Surveillance Programme (EGASP), Thailand, 2015–2021

**DOI:** 10.1371/journal.pone.0305296

**Published:** 2024-07-05

**Authors:** Jaray Tongtoyai, Thitima Cherdtrakulkiat, Natnaree Girdthep, Silvina Masciotra, Santi Winaitham, Pongsathorn Sangprasert, Ekkachai Daengsaard, Anuparp Puangsoi, Rossaphorn Kittiyaowamarn, Eileen F. Dunne, Pachara Sirivongrangson, Andrew C. Hickey, Emily Weston, Rebekah M. Frankson

**Affiliations:** 1 Division of HIV Prevention, U.S. Centers for Disease Control and Prevention, Atlanta, Georgia, United States of America; 2 Thailand Ministry of Public Health, U.S. Centers for Disease Control and Prevention Collaboration, Nonthaburi, Thailand; 3 Department of Disease Control, Thailand Ministry of Public Health, Nonthaburi, Thailand; 4 Division of STD Prevention, U.S. Centers for Disease Control and Prevention, Atlanta, Georgia, United States of America; University of Cape Coast, GHANA

## Abstract

**Background:**

Quality assessments of gonococcal surveillance data are critical to improve data validity and to enhance the value of surveillance findings. Detecting data errors by systematic audits identifies areas for quality improvement. We designed and implemented an internal audit process to evaluate the accuracy and completeness of surveillance data for the Thailand Enhanced Gonococcal Antimicrobial Surveillance Programme (EGASP).

**Methods:**

We conducted a data quality audit of source records by comparison with the data stored in the EGASP database for five audit cycles from 2015–2021. Ten percent of culture-confirmed cases of *Neisseria gonorrhoeae* were randomly sampled along with any cases identified with elevated antimicrobial susceptibility testing results and cases with repeat infections. Incorrect and incomplete data were investigated, and corrective action and preventive actions (CAPA) were implemented. Accuracy was defined as the percentage of identical data in both the source records and the database. Completeness was defined as the percentage of non-missing data from either the source document or the database. Statistical analyses were performed using the t-test and the Fisher’s exact test.

**Results:**

We sampled and reviewed 70, 162, 85, 68, and 46 EGASP records during the five audit cycles. Overall accuracy and completeness in the five audit cycles ranged from 93.6% to 99.4% and 95.0% to 99.9%, respectively. Overall, completeness was significantly higher than accuracy (p = 0.017). For each laboratory and clinical data element, concordance was >85% in all audit cycles except for two laboratory data elements in two audit cycles. These elements significantly improved following identification and CAPA implementation.

**Discussion:**

We found a high level of data accuracy and completeness in the five audit cycles. The implementation of the audit process identified areas for improvement. Systematic quality assessments of laboratory and clinical data ensure high quality EGASP surveillance data to monitor for antimicrobial resistant *Neisseria gonorrhoeae* in Thailand.

## Introduction

Data quality is a cornerstone of public health surveillance as data are used to analyze, plan, implement, and evaluate public health issues and practice [1, 2]. Poor data quality can lead to errors in the interpretation of data, and decrease the usefulness, accuracy, and reliability of the surveillance system. Poor data quality can ultimately delay the achievement of public health program goals and objectives [2, 3].

Documentation of steps taken to ensure the highest data quality possible, such as through a quality management system (QMS), provides a historical record that the best standard of care was provided to patients [4, 5] and provides transparent quality assurance measures for surveillance programs. A QMS is a formalized and comprehensive system that documents processes, procedures, and responsibilities to achieve quality policies and objectives through continual improvement. An important component of QMS is documentation, specifically capturing complete, accurate data, maintaining proper documentation (including documentation such as protocols and standard operating procedures [SOPs]), and performing routine evaluations and internal audits of data [6]. Implementing internal audits and documentation review of surveillance data aims to assess, detect data errors or nonconformity, and can help direct corrective action and preventive actions (CAPA) leading to stronger and more reliable surveillance systems [7, 8].

Antimicrobial resistant (AMR) *Neisseria gonorrhoeae* is a global public health threat as the extended spectrum cephalosporins are currently the last recommended empirical treatment options for *N*. *gonorrhoeae*. Additionally, in the past few years, more countries have started to report cefixime and ceftriaxone (both cephalosporin class antibiotics) resistant *N*. *gonorrhoeae* isolates [9–12]. As a result, surveillance for AMR *N*. *gonorrhoeae* is needed to monitor trends of resistance [13–15]. The World Health Organization (WHO) implemented the Gonococcal Antimicrobial Surveillance Programme (GASP) in 1990 to monitor AMR *N*. *gonorrhoeae* worldwide [16]. In 2009, WHO strengthened the program by conducting external quality assessments (EQA) and distributing WHO *N*. *gonorrhoeae* reference strains for internal quality control [17]. In 2012, WHO released a global action plan which laid out steps for the control and spread of AMR gonorrhea; one key component of the plan was strengthening AMR surveillance [18, 19]. As a result, in late 2015 the Thailand Ministry of Public Health (MOPH), WHO, and United States Centers for Disease Control and Prevention (U.S. CDC) implemented the Enhanced Gonococcal Antimicrobial Surveillance Programme (EGASP) to monitor trends in antimicrobial susceptibilities in *N*. *gonorrhoeae*. Like GASP, EGASP collects isolates from cases diagnosed with *N*. *gonorrhoeae* for antimicrobial susceptibility testing. However, EGASP also aims to collect clinical, epidemiological, and behavioral data such as demographics, sexual behavior history, antibiotic use, and treatment information using standardized methodologies [20].

EGASP surveillance data have already impacted local and national policy in Thailand. In 2019, the Thailand MOPH used local EGASP data to update the treatment guidelines for gonorrhea and non-gonococcal urethritis [21]. Due to rising minimum inhibitory concentrations (MIC) for cefixime and ceftriaxone seen among EGASP isolates over time, the Thailand MOPH changed the guidance for gonorrhea treatment from a single dose of 250 mg ceftriaxone intramuscular (IM) to 500 mg ceftriaxone IM single dose [21]. In response to enhanced surveillance activity, Thailand MOPH updated sexually transmitted infections screening guidelines in 2022 by adding *N*. *gonorrhoeae* culture for individuals at risk and with recent sexual contact (within one week following sexual contact [22]. The EGASP Thailand data continue to be used to inform the national strategy for gonorrhea clinical management. As such, we aimed to develop and evaluate a reliable data quality assessment approach to assure accuracy and completeness of the pilot program’s surveillance data. Further, we aimed to evaluate the impact on data quality through five of the quality audit cycles of the paired audit and corrective action approach.

## Materials and methods

### EGASP surveillance data collection

Bangrak Hospital (BH) and Silom Community Clinic@TropMed (SCC) were the only two sentinel sites included in Thailand EGASP until 2022. BH is the largest sexually transmitted infections (STIs) center in Bangkok. It is a public clinic that provides STI diagnosis and treatment for the general population, which includes cisgender women, transgender women (TGW) and cisgender men, including men who have sex with men (MSM). SCC is a clinic in central Bangkok that conducts clinical research and provides voluntary human immunodeficiency virus (HIV) and STI counseling and testing primarily for MSM and TGW clients. At both BH and SCC, in addition to the collection of urethral specimens from cisgender men presenting symptoms of urethritis (i.e., discharge or dysuria), select demographic, behavioral, and clinical data were obtained.

**Fig 1:**
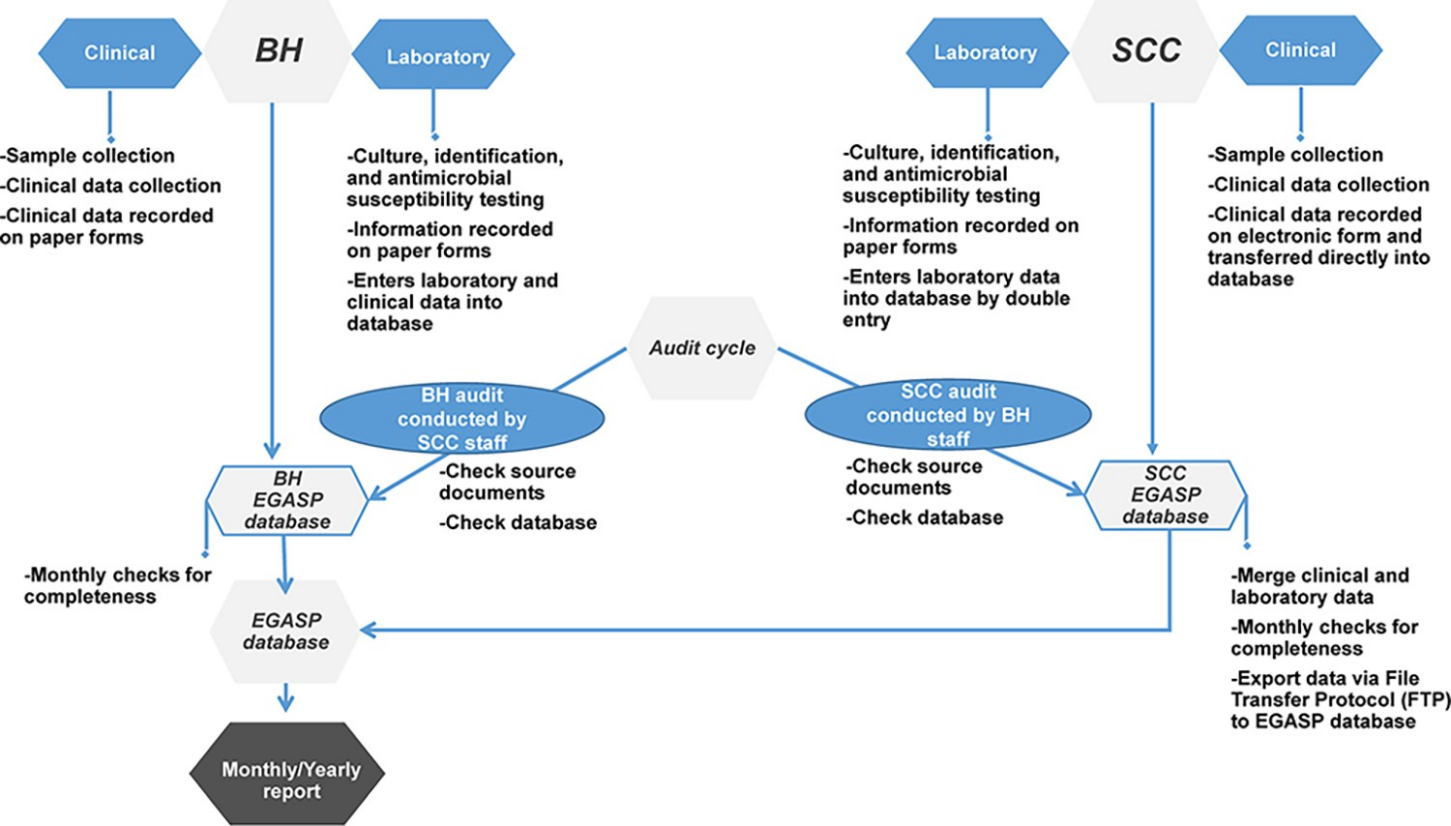
Overall data flow, EGASP Thailand, 2015–2021. Diagram showing the pathway for clinical and laboratory data in this surveillance pilot program, including the data audit approach for Bangrak Hospital (BH) and Silom Community Clinic (SCC).

A unique EGASP identification (ID) number was assigned to each case and manually linked to the laboratory, demographic, behavioral, and clinical data (but not to personally identifiable information). Laboratory methods, including Gram stain, culture, and antimicrobial susceptibility testing, were performed following approved SOPs, and the results were recorded manually on paper forms. Briefly, for *N*. *gonorrhoeae* culture and antimicrobial susceptibility testing, the urethral specimen was inoculated on Modified Thayer-Martin media plate (in-house media preparation following good quality control practice; Oxoid, Basingstoke, UK). The inoculated plate was incubated at 35°C–36.5°C for up to 48 hours in a humid chamber (70–80%) supplemented with 5% CO_2_. Typical-appearing colonies were confirmed using the rapid carbohydrate utilization test (in-house preparation following good laboratory quality control practice; BD Difco, Sparks, MD, USA; KEMAUS, Cherrybrook, N.S.W, Australia). *N*. *gonorrhoeae* isolates were stored at –70°C in a suitable cryopreservation media (10% skim milk containing 10% glycerol prepared following good quality control techniques) until antimicrobial susceptibility testing. Antibiotic MICs for *N*. *gonorrhoeae* isolates were determined using Etest (bioMérieux, Marcy-l’Étoile, France) according to manufacturer’s instructions [20] according to guidelines from the Clinical and Laboratory Standards Institute (CLSI) guidelines (Performance Standards for Antimicrobial Susceptibility Testing M100-S24) [23]. All behavioral and clinical data, such as age, prior antibiotic use, sexual behavior history, and treatment were considered core elements for EGASP and were collected on a standardized abstraction form. Data management at both sites followed a standardized data management SOP. All paper-based source records were entered into the EGASP electronic database. Data entry was checked monthly for completeness by surveillance and data personnel at each site. Cleaned data from the two sites were merged, and a monthly standardized report was generated and presented at routine team meetings and further disseminated to AMR *N*. *gonorrhoeae* Surveillance Programs in Thailand MOPH, WHO and U.S. CDC colleagues involved with EGASP management (Fig 1).

### Data audit and review

Each audit included four key steps: planning, assessment, reporting, and follow-up. During the initial planning phase, a data audit SOP was created that included sampling methodology, an audit timeline, and the laboratory and clinical data checklist forms. Following the training of surveillance personnel, staff dedicated to this activity conducted five separate cycles of data audits covering the following periods during November 2015 through November 2021 (S1 Table): Cycle 1 (September 2016 assessing data collected during November 2015 through June 2016), Cycle 2 (June 2018 assessing data collected during July 2016 through November 2017), Cycle 3 (April 2019 assessing data collected during December 2017 through October 2018), Cycle 4 (June 2020 assessing data collected during November 2018 through October 2019) and Cycle 5 (November 2021 assessing data collected during November 2019 through December 2020).

For the assessment, the audit for each sentinel site was performed by staff from the other sentinel site; that is, BH staff reviewed data from SCC and SCC staff reviewed data from BH. The audit sample size included a random selection of 10% [24, 25] of EGASP *N*. *gonorrhoeae* culture-confirmed cases (except for Cycle 1 in which 10% of all EGASP records were randomly selected) as well as all cases with an MIC alert value (i.e., MICs above a defined threshold) and all cases with repeat *N*. *gonorrhoeae* infection. Audited data elements included the core surveillance elements submitted monthly to Thailand MOPH, WHO, and U.S. CDC. The laboratory data checklist form consisted of 15 data elements: EGASP ID number, patient visit date, urethral Gram stain result (double-checked against the clinical Gram stain result), *N*. *gonorrhoeae* culture identification result, isolate storage date, recorded antimicrobial susceptibility testing results for five tested antibiotics (azithromycin, cefixime, ceftriaxone, ciprofloxacin, and gentamicin), and recorded MIC alert values. The clinical data checklist form consisted of eight data elements: EGASP ID number, patient visit date, gender of sexual partner, prior antibiotic use, urethral Gram stain result as reported from the clinic, diagnosis, and treatment.

Elements from the original source documents (i.e., laboratory records, clinical abstraction forms) were compared to the equivalent data recorded in the EGASP database. All measures were dichotomous (yes/no). Elements were deemed concordant (and coded “yes”) if the reviewer determined that the data element from the source document matched the data recorded in the EGASP database and that there were no missing data in either the source document or the EGASP database. Elements were deemed discordant (and coded “no”) if either the reviewer determined the data element was a mismatch or was missing from either the source document or the EGASP database.

After each audit cycle, the accuracy and completeness of laboratory and clinical records were reviewed by surveillance personnel to help identify steps, processes, or documents/SOPs that needed improvement and/or clarification. CAPA were implemented after each cycle to improve data quality. The findings of each audit cycle and corresponding CAPA results were presented during monthly EGASP team meetings.

### Data analysis

Data were analyzed using IBM SPSS version 22.0 (IBM Corp., Armonk, N.Y., USA). Concordance was calculated as the number of identical and complete data elements in the source document and the EGASP database divided by the total number of records reviewed. Accuracy was defined and calculated as the number and percentage of identical data in both the source document and the EGASP database. Completeness was defined as the number and percentage of non-missing data from either the source document or the EGASP database. Overall accuracy and completeness were the number and percentage of all identical elements with no missing data in both laboratory and clinical data, divided by the total number of elements. Statistical analyses comparing Cycle 1 and Cycle 2, Cycle 2 and Cycle 3, Cycle 3 and Cycle 4, and Cycle 4 and Cycle 5 were performed using Fisher’s exact test for a 2x2 contingency table (i.e., concordance data of each data element, and accuracy and completeness of laboratory and clinical data). The odds ratio (OR) and associated confidence interval (CI), were calculated as the ratio of concordance data from two audit cycles. A paired sample t-test was applied to evaluate the difference between overall accuracy and overall completeness outcomes; p<0.05 was considered statistically significant.

## Results

A summary of urethritis and *N*. *gonorrhoeae* cases identified during the EGASP pilot surveillance program between November 2015 through December 2020 are provided in S1 Table. Briefly, 151 and 78 cases (2015); 1103 and 613 cases (2016); 644 and 376 cases (2017); 587 and 358 cases (2018); 601 and 250 cases (2019); as well as 407 and 143 cases (2020) of urethritis and *N*. *gonorrhoeae*, respectively, were observed each year, with 1818 total *N*. *gonorrhoeae* infections identified during the full surveillance period. Among the 1818 *N*. *gonorrhoeae* individual isolates obtained, none exhibited a resistance phenotype exceeding the established surveillance program breakpoint (MIC >0.25 μg/mL) for first-line treatments (according to Thai national guidelines) [21, 26], specifically ceftriaxone.

The following number of records from EGASP cases were chosen for each audit cycle: 70 of 699 (10.0%) records in Cycle 1; 162 of 656 (24.7%) records in Cycle 2; 85 of 399 (21.3%) records in Cycle 3; 68 of 620 (10.9%) records in Cycle 4; and 46 of 168 (27.4%) records in Cycle 5 (S1 Table). Five clinical source documents from Cycle 2 and four clinical source documents from Cycle 5 became missing when BH relocated between May 2017 and January 2021 so were excluded from the analysis of clinical accuracy and completeness. Therefore, 157 and 42 clinical source documents and 162 and 46 laboratory records from Cycle 2 and Cycle 5, respectively, were included in the analysis. EGASP documentation were assessed separately for two aspects, accuracy and completeness, for laboratory, clinical, and overall (combined) program records.

**Fig 2:**
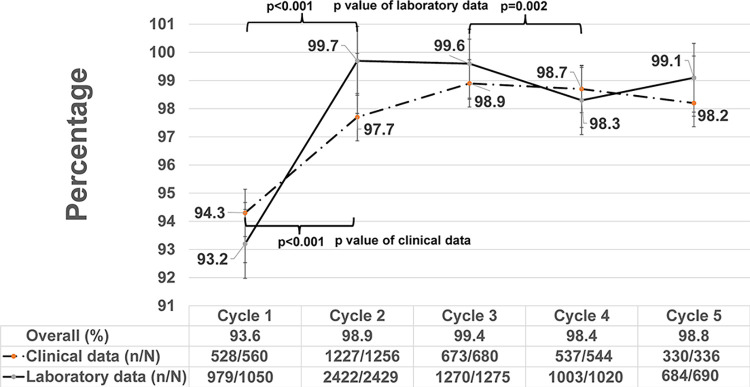
Accuracy of laboratory and clinical data by data audit cycle, EGASP Thailand, 2015–2021. Comparison of source document and database accuracy over the course of the pilot surveillance project from 2015–2020 (Cycle 1: 11/2015–6/2016, Cycle 2: 7/2016–11/2017, Cycle 3: 12/2017–10/2018, Cycle 4: 11/2018–10/2019, and Cycle 5: 11/2019–12/2020). Accuracy was calculated as the proportion of data elements from both source documents and surveillance database that were identical (n) compared to all elements reviewed during the audit (N), for data from the clinical encounter (clinical data), laboratory workup (laboratory data), and clinical/laboratory combined (overall). The trend in accuracy of clinical (dashed line) and laboratory (solid line) data are shown over the surveillance period (cycles 1–5) with 95% confidence intervals (vertical bars).

**Fig 3:**
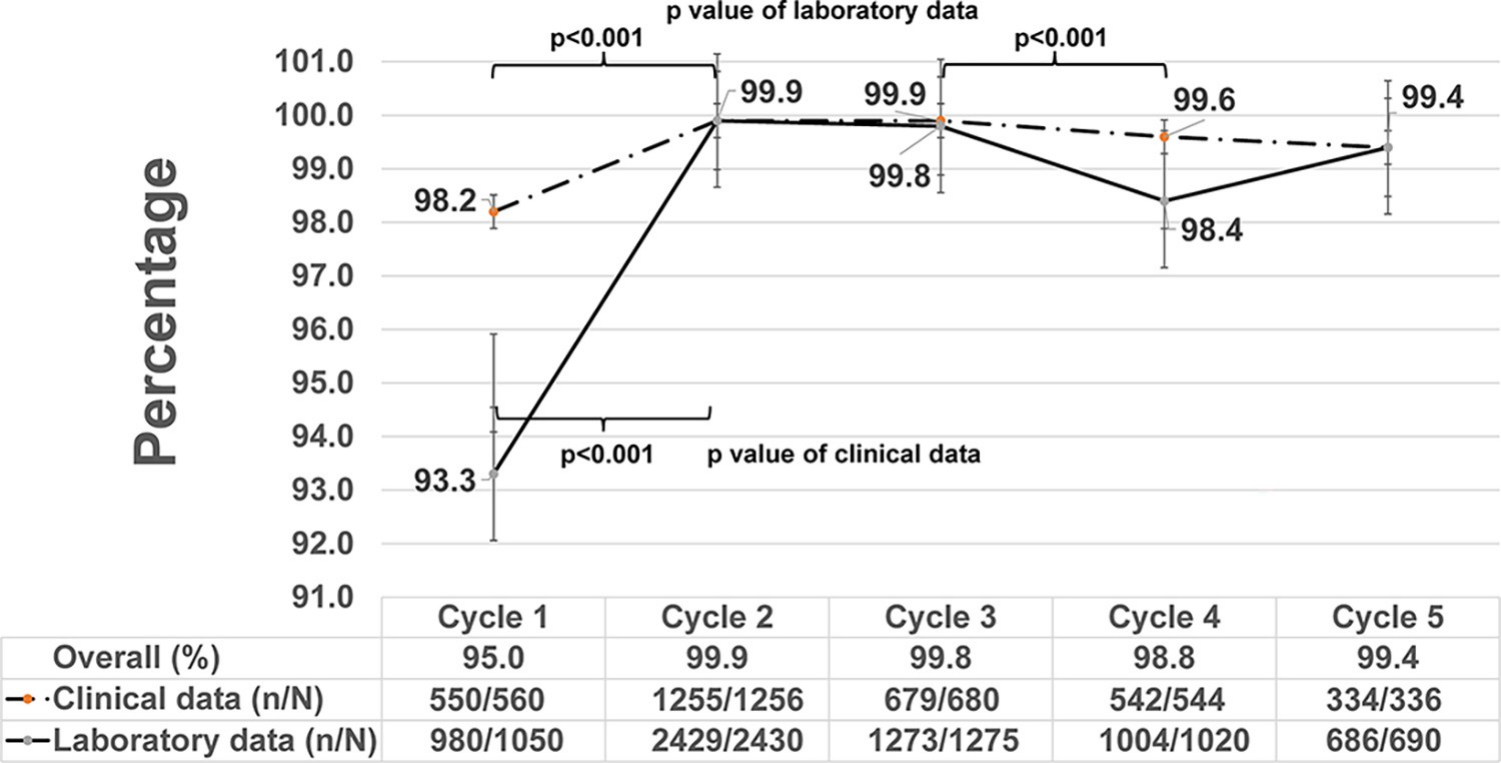
Completeness of laboratory and clinical data by data audit cycle, EGASP Thailand, 2015–2021. Comparison of source document and database completeness over the course of the pilot surveillance project from 2015–2020 (Cycle 1: 11/2015–6/2016, Cycle 2: 7/2016–11/2017, Cycle 3: 12/2017–10/2018, Cycle 4: 11/2018–10/2019, and Cycle 5: 11/2019–12/2020). Completeness was calculated as the proportion of data elements from source documents and surveillance database that were complete (no missing data in either source documents or database, n) compared to all elements reviewed during the audit (N), for data from the clinical encounter (clinical data), laboratory workup (laboratory data), and clinical/laboratory combined (overall). The trend in completeness of clinical (dashed line) and laboratory (solid line) data are shown over the surveillance period (cycles 1–5) with 95% confidence intervals (vertical bars).

Overall accuracy for laboratory and clinical data elements ranged from 93.6% to 99.4% (Fig 2). The greatest increase in overall accuracy occurred between Cycle 1 and Cycle 2 (p<0.001), likely resulting from standardization of practices, improved familiarity with the program and processes, and better comprehension/standardization of documentation procedures following the initial audit. Accuracy and completeness of EGASP laboratory and clinical data over five audit cycles are shown in Figs 2 and 3, respectively. Laboratory data accuracy ranged from 93.2% to 99.7%, mirroring overall accuracy trends, with an observed significant increase from 93.2% in Cycle 1 to 99.7% in Cycle 2 (p<0.001, Fig 2). Laboratory data accuracy remained high over the entire course of the audit periods, though significant differences were observed between cycles (Fig 2). Clinical data accuracy ranged from 94.3% to 98.9%, again with a significant increase in accuracy between Cycle 1 (94.3%) and Cycle 2 (97.7%, p<0.001, Fig 2) and subsequently remained above 98.2%.

Overall completeness for laboratory and clinical data elements ranged from 95.0% to 99.9% (Fig 3), with significant improvement in overall completeness between Cycle 1 and Cycle 2 (p<0.001) and consistently high completeness thereafter. Trends in completeness of laboratory and clinical data were similar to those observed for data accuracy (Fig 3). Overall, laboratory and clinical data were complete, but accuracy of laboratory and clinical data was suboptimal during data entry into the EGASP database as indicated by the results of the t-test analysis (p = 0.017).

To identify specific areas that needed improvement during the data collection process, the concordance for each data element included in the laboratory and clinical audit forms was assessed. Concordance expressed in percentage for the laboratory and clinical data elements of each audit cycle, and the results of the statistical analysis of the comparison between cycles (p values) are reported in Tables 1 and 2.

**Table 1:** Concordance of laboratory data elements between source documents and EGASP database for five audit cycles, EGASP Thailand, 2015–2021.

Data Characteristics	Concordance %					*Cycle-1/2*		*Cycle-2/3*		*Cycle-3/4*		*Cycle-4/5*	
	Cycle-1	Cycle-2	Cycle-3	Cycle-4	Cycle-5	OR (CI)	Fisher exact P-value	OR (CI)	Fisher exact P-value	OR (CI)	Fisher exact P-value	OR (CI)	Fisher exact P-value
	(N = 70)	(N = 162)	(N = 85)	(N = 68)	(N = 46)								
1. EGASP number	100	100	100	98.5	100	1.006 (0.994, 1.018)	1.000	0.994 (0.982, 1.006)	1.000	1.015 (0.986, 1.045)	0.444	0.985 (0.957, 1.014)	1.000
2. Visit Date (DD/MM/YYYY)	100	99.4	98.8	100	100	1.006 (0.994, 1.018)	1.000	1.006 (0.980, 1.032)	1.000	0.988 (0.966, 1.011)	1.000	N/A***	1.000
3. Gram stain result from urethral swab (at clinic)	100	99.4	100	100	100	1.006 (0.994, 1.018)	1.000	0.994 (0.982, 1.006)	1.000	N/A***	1.000	N/A***	1.000
4. Morphologically typical colonies	50	100	100	100	97.8	0.500 (0.396, 0.632)	<0.001	N/A***	1.000	N/A***	1.000	1.022 (0.979, 1.067)	0.404
5. Final Identification	100	100	100	100	87.4	N/A***	1.000	N/A***	1.000	N/A***	1.000	1.070 (0.991, 1.155)	0.063
6. Isolation storage date	100	96.9	96.5	100	100	1.032 (1.004, 1.061)	0.326	1.005 (0.956, 1.005)	1.000	0.965 (0.926, 1.005)	0.254	N/A***	1.000
7. NG Isolates available for AST after re-culture from freezing media (growth, no growth, contaminate)	51.4	100	98.8	76.5	100	0.514 (0.410, 0.646)	<0.001	1.012 (0.989, 1.036)	0.344	1.292 (1.130, 1.477)	<0.001	0.765 (0.670, 0.872)	<0.001
8. CRO value	100	100	100	100	97.8	N/A***	1.000	N/A***	1.000	N/A***	1.000	1.022 (0.979, 1.067)	0.404
9. CFM value	100	100	100	100	100	N/A***	1.000	N/A***	1.000	N/A***	1.000	N/A***	1.000
10. AZI value	100	100	100	100	100	N/A***	1.000	N/A***	1.000	N/A***	1.000	N/A***	1.000
11. GEN value	100	100	100	100	97.8	N/A***	1.000	N/A***	1.000	N/A***	1.000	1.022 (0.979, 1.067)	0.404
12. CIP value	100	99.4	100	100	100	1.006 (0.994, 1.018)	1.000	0.994 (0.982, 1.006)	1.000	N/A***	1.000	N/A***	1.000
13. AST report date	100	100	100	100	100	N/A***	1.000	N/A***	1.000	N/A***	1.000	N/A***	1.000
14. Initial alert date/by	98.6	100	100	100	100	0.986 (0.958, 1.014)	0.302	N/A***	1.000	N/A***	1.000	N/A***	1.000
15. Repeat initial alert tests within 5 working days	98.6	100	100	100	100	0.986 (0.958, 1.014)	0.302	N/A***	1.000	N/A***	1.000	N/A***	1.000

Abbreviations: N = total number of reviewed elements, ID = identification, NG = *Neisseria gonorrhoeae*, AST = Antimicrobial Susceptibility Testing, AZI = Azithromycin, CFM = Cefixime, CRO = Ceftriaxone, CIP = Ciprofloxacin, GEN = Gentamicin, OR = Odds ratio, CI = Confidence interval

***No statistics are computed; variable is a constant.

Data from each cycle were collected in the following time periods: Cycle 1 (11/2015–6/2016), Cycle 2 (7/2016–11/2017), Cycle 3 (12/2017–10/2018), Cycle 4 (11/2018–10/2019) and Cycle 5 (11/2019–12/2020)

**Table 2:** Concordance of clinical data elements between source documents and EGASP database for five audit cycles, EGASP Thailand, 2015–2021.

Data Characteristics	Concordance (%)					*Cycle-1/2*		*Cycle-2/3*		*Cycle-3/4*		*Cycle-4/5*	
	Cycle-1	Cycle-2 *	Cycle-3	Cycle-4	Cycle-5	OR (CI)	Fisher exact P-value	OR (CI)	Fisher exact P-value	OR (CI)	Fisher exact P-value	OR (CI)	Fisher exact P-value
	(N = 70)	(N = 157)	(N = 85)	(N = 68)	**(N = 42)								
1. EGASP number	100	100	98.8	100	100	N/A***	1.000	1.012 (0.989, 1.036)	0.351	0.988 (0.966, 1.011)	1.000	N/A***	1.000
2. Visit Date (DD/MM/YYYY)	100	99.4	98.8	100	100	1.006 (0.994, 1.019)	1.000	1.005 (0.979, 1.032)	1.000	0.988 (0.966, 1.011)	1.000	N/A***	1.000
3. Gender of sex partner	90	94.9	98.8	98.5	95.2	0.948 (0.870, 1.034)	0.245	0.960 (0.920, 1.003)	0.166	1.003 (0.966, 1.041)	1.000	1.035 (0.961, 1.114)	0.557
4. Antibiotic use in the previous 2 weeks	100	96.8	97.6	98.5	95.2	1.033 (1.004, 1.063)	0.327	0.991 (0.949, 1.036)	1.000	0.991 (0.948, 1.036)	1.000	1.035 (0.961, 1.114)	0.557
5. Urethral gram stain result (only GNID)	97.1	99.4	100	100	100	0.978 (0.937, 1.020)	0.226	0.994 (0.981, 1.006)	1.000	N/A***	1.000	N/A***	1.000
6. Diagnosis at current visit	95.7	93.6	97.6	97.1	100	1.022 (0.959, 1.090)	0.759	0.959 (0.910, 1.011)	0.223	1.006 (0.954, 1.061)	1.000	0.971 (0.931, 1.012)	0.524
7. Primary treatment for gonorrhea	85.7	99.4	100	98.5	97.6	0.863 (0.783, 0.950)	<0.001	0.994 (0.981, 1.006)	1.000	1.015 (0.986, 1.045)	0.444	1.009 (0.955, 1.067)	1.000
8. Presence of dual treatment for gonorrhea	85.7	98.1	100	97.1	97.6	0.874 (0.792, 0.964)	0.001	0.981 (0.960, 1.003)	0.554	1.030 (0.989, 1.074)	0.196	0.994 (0.934, 1.059)	1.000

Abbreviations: N = total number of reviewed elements, ID = identification, GNID = gram negative intracellular diplococci, OR = Odds ratio, CI = Confidence interval

*Due to missing source documents in the clinical data, the number of clinical/epidemiology data records reviewed in Cycle 2 was 157 and the number of laboratory records reviewed was 162.

**Due to missing source documents in the clinical data, the number of clinical/epidemiology data records reviewed in Cycle 5 was 42 and the number of laboratory records reviewed was 46.

***No statistics are computed; variable is a constant.

Data from each cycle were collected in the following time periods: Cycle 1 (11/2015–6/2016), Cycle 2 (7/2016–11/2017), Cycle 3 (12/2017–10/2018), Cycle 4 (11/2018–10/2019) and Cycle 5 (11/2019–12/2020)

Among fifteen laboratory data elements, thirteen had concordance ≥87.4% in all five cycles; however, only 50% and 51.4% concordance were found for the “morphologically typical colonies” and “*N*. *gonorrhoeae* isolates available for antimicrobial susceptibility testing after re-culture from freezing media” elements, respectively, for Cycle 1 (Table 1). In addition to discordance among those two laboratory elements, 69 of 1050 laboratory data from 36 of 70 records reviewed in the EGASP database did not have a supporting source document in Cycle 1. Investigation of the identified nonconformity showed that inappropriate laboratory data collection sheets were being used at the BH site when recording data. The CAPA process resulted in updating the EGASP laboratory data collection sheets. Implementation of the revised laboratory data collection process resulted in a statistically significant improvement in the concordance of both elements to 100% (p<0.001) in Cycle 2. However, concordance for the “*N*. *gonorrhoeae* isolates available for antimicrobial susceptibility testing after re-culture from freezing media” element significantly decreased in Cycle 4. After investigation, it was found that new laboratory staff had not followed the laboratory SOP. To prevent recurrence, all laboratory staff were retrained and more closely supervised by senior laboratory staff during a monitoring period of a few months to ensure full understanding of the laboratory SOP and as a result, concordance for this data element was 100% in Cycle 5 (p<0.001, Table 1).

Among eight clinical data elements, six had >90% concordance in all five cycles. Concordance for the “primary treatment for gonorrhea” and “presence of dual treatment for gonorrhea” elements had 85.7% concordance in Cycle 1, which significantly improved (p≤0.001) in Cycle 2 (Table 2). The investigation identified data entry errors of these elements in the abstraction form due to the misunderstanding of the variable itself. CAPA implementation included revision of the data abstraction form and staff re-training. The Cycle 2 audit found an improvement in concordance to >98.1%. In Cycle 5, no significant decrease in concordance was noted for some laboratory and clinical data elements (Tables 1 and 2). The investigation identified the possible causes were random human error and less adherence to follow SOPs.

## Discussion

Our EGASP Thailand quality assessment showed that the two sentinel sites reported high quality surveillance data. Overall accuracy and completeness for the five audit cycles were high. Based on these data audits, gaps were identified, corrective actions were implemented, and the results show that these corrective actions supported improvements in overall data quality.

The accuracy and completeness of laboratory and clinical data significantly improved in Cycle 2 after corrective actions were implemented; high accuracy and completeness were maintained throughout the remaining audit cycles except for Cycle 4. Overall completeness was found to be significantly higher than overall accuracy. However, laboratory completeness was slightly lower than clinical completeness in Cycle 1 and Cycle 4. Although data completeness was checked monthly at both sentinel sites before merging data for the preparation of the monthly report, completeness checks were only performed on recorded data in the EGASP database, but not on the data in the paper source documents. EGASP data were sometimes entered into the EGASP database without a supporting source document. These findings are frequently found when conducting an audit [27] and highlight the importance of comparing data in both the source documents and the main database during a data audit [28].

The audit process also became an opportunity to clarify procedures and data collection misunderstandings and update SOPs as necessary. Concordance between data in the original source documents and data in the EGASP database was >90% for most laboratory and clinical elements, except for two laboratory elements and two clinical elements in Cycle 1. Audits allowed the surveillance team to identify the incorrect use of inappropriate laboratory collection sheets and poor compliance to follow the SOP by new staff. Correction of these errors resolved the issues to achieve high surveillance quality by the following audit and further prevent recurrence of issues in future cycles in most cases. These findings demonstrate the importance of routine audits to identify issues throughout the course of surveillance implementation, but also to identify problems that may arise over time as staffing changes. The clarity of the record forms and the quality of training and re-training are influential factors to help to improve data quality [2].

The results also highlight the importance of following the SOPs, re-training, and clear communication with staff about SOPs to solve nonconformity or deviations [29]. QMS standards recommend providing staff with an SOP that describes clear step-by-step instructions to improve consistency among staff [30, 31]. Some nonconforming events were identified during the EGASP audits which required the implementation of preventive actions. Data collection for EGASP Thailand relied heavily on paper forms which then needed to be data entered into the in-house electronic database. This process can result in human transcription errors [32], and an increased workload burden for staff. Data collection in a standardized, electronic platform such as an internet-based system [33] may be an option to reduce workload and human data transcription errors [32], ultimately allowing for more timely analysis of data [34, 35].

Audit Cycle 4 was performed during the pandemic in June 2020 and Cycle 5 in November 2021, 17 months later. Thailand implemented alternative working schedules to maintain the safety of staff during the COVID pandemic and as a result, overall compliance to SOPs may have decreased due to increased workloads when on-site. In addition, the BH sentinel site was relocated to a newly remodeled and updated location in 2021 after being housed in a temporary campus. During the audit process, we found that original source documents were missing and as a result, some clinical source documents were not able to be included in the audits. The misplacing of source documents during the first move to temporary quarters, which was not thoroughly investigated, and CAPA not being implemented were likely to be the causes of poor document/data management during the return to the remodeled spaces [36]. The trend of reduced concordance in Cycle 5 compared to previous cycles may be a result of the impact of the COVID pandemic and the temporary relocation of one sentinel site for remodeling and updates to the facility.

The EGASP audit procedure we instituted had several limitations. It was labor intensive and involved numerous team members at multiple sites; scheduling audit activities in addition to routine on-site work was difficult and may not be feasible in all settings. Modifications to the data quality audit procedure may improve the process of obtaining high quality data. For example, implementation of an electronic system to capture clinical data with internal checks for completeness might decrease the time required to review records for an annual audit. In addition, some audits were performed more than one year after data collection and thus might not have identified data quality issues prior to reporting data to partners. More frequent reviews, such as quarterly reviews, would help to ensure quality data in a timely manner [3, 24]. Internal audit within sentinel sites may be suitable for this recommendation. Moreover, this quality assessment was performed with an arbitrary subset of records from EGASP cases as defined in the planning steps and may not be representative. Regular review and careful consideration of the algorithm for data auditing may be needed to identify issues and/or address systemic and programmatic issues (e.g., consistently missing data, staff turnover, and the implementation of new procedures and/or SOPs). The cause of the missing source documents was not thoroughly investigated due to missing source documentation. Appropriate modifications to SOPs to prevent the loss of source documentation would facilitate improved audits and investigation as well as contributing to improved data assurance. Lastly, a thorough data quality assessment should assess not only accuracy and completeness, but also timeliness, feasibility, generalizability, and other attributes such as accessibility and consistency [3].

The successful implementation of EGASP in the capital, Bangkok, of Thailand, prompted recent expansions of Thailand’s EGASP to popular tourism destinations for travelers. During October 2022, enhanced gonococcal surveillance activities were established in Chiang-Mai province (northern Thailand), followed by Chonburi province (eastern Thailand) in June 2023. Additional expansions of the program are being considered to improve EGASP surveillance representativeness for the whole of Thailand.

## Conclusion

Systematic quality assessments of laboratory and clinical data ensure high quality EGASP surveillance data to monitor for AMR *N*. *gonorrhoeae* in Thailand. Our quality assessment of Thailand EGASP surveillance data improved the data collection process, helped maintain high-quality data generation from two sentinel sites, and confirmed the accuracy of *N*. *gonorrhoeae* drug resistant surveillance data. The results highlight the importance of implementing quality assessments of surveillance data, especially at early stages of program implementation. Tools and processes for quality assessments, such as SOPs, training and refresher-training for staff, and standardized forms for data collection and audit, are critical elements of a quality management system to strengthen surveillance data by improving data quality.

## Supporting information

S1 Table EGASP cases and *N*. *gonorrhoeae* cases divided by calendar year and EGASP data overlapping of audit cycle, Thailand, 2015–2020. (PDF)

S2 Table Laboratory data. (PDF)

S3 Table Clinical data. (PDF)

S4 Table Overall accuracy and completeness. (PDF)
