# Supplementary material for: Data quality assessment of the Enhanced Gonococcal Antimicrobial Surveillance Programme (EGASP), Thailand, 2015–2021
Source: PLoS One. 2024 Jul 5;19(7):e0305296. doi: 10.1371/journal.pone.0305296 (PMC11226028; doi:10.1371/journal.pone.0305296)
Supplement: S1 Table — (PDF) [file pone.0305296.s001.pdf]

S1 Table. EGASP cases and *N. gonorrhoeae* cases divided by calendar year and EGASP data overlapping of audit cycle, Thailand, 2015 – 2020.

Year/month	2015	2016	2017	2018	2019	2020	Total Cases
	11 12	1 2 3 4 5 6 7 8 9 10 11 12	1 2 3 4 5 6 7 8 9 10 11 12	1 2 3 4 5 6 7 8 9 10 11 12	1 2 3 4 5 6 7 8 9 10 11 12	1 2 3 4 5 6 7 8 9 10 11 12	
**Urethritis (EGASP) cases/Site/Year (BH/SCC)**	132/19	838/265	431/213	466/121	499/102	345/62	3493
**Total urethritis (EGASP)/Year**	151	1103	644	587	601	407	
***N. gonorrhoeae* cases/site/Year(BH/SCC)**	69/9	494/119	276/100	290/68	196/54	115/28	1818
**Total *N. gonorrhoeae* cases/Year**	78	613	376	358	250	143	

Audit cycle	Cycle 1	Cycle 2	Cycle 3	Cycle 4	Cycle 5	
**Number of audited cases**	70	162	85	68	46	431
**Month of audit**	September 2016	June 2018	April 2019	June 2020	November 2021	

Abbreviations: BH = Bangrak Hospital, SCC= Silom Community Clinic@TropMed,
