# Supplementary material for: Data quality assessment of the Enhanced Gonococcal Antimicrobial Surveillance Programme (EGASP), Thailand, 2015–2021
Source: PLoS One. 2024 Jul 5;19(7):e0305296. doi: 10.1371/journal.pone.0305296 (PMC11226028; doi:10.1371/journal.pone.0305296)
Supplement: S2 Table — (PDF) [file pone.0305296.s002.pdf]

S2 Table. Laboratory data.

Data Characteristics	Discordance n/N				
	Cycle-1 (N= 70)	Cycle-2 (N=162)	Cycle-3 (N=85)	Cycle-4 (N=68)	Cycle-5 (N= 46)
1. EGASP number	0	0	0	1	0
2. Visit Date (DD/MM/YYYY)	0	1	1	0	0
3. Gram stain result from urethral swab (at clinic)	0	1	0	0	0
4. Morphologically typical colonies	35	0	0	0	1
5. Final Identification	0	0	0	0	3
6. Isolation storage date	0	5	3	0	0
7. NG Isolates available for AST after re-culture from freezing media (growth, no growth,	34	0	1	16	0
8. CRO value	0	0	0	0	1
9. CFM value	0	0	0	0	0
10. AZI value	0	0	0	0	0
11. GEN value	0	0	0	0	1
12. CIP value	0	1	0	0	0
13. AST report date	0	0	0	0	0
14. Initial alert date/by	1	0	0	0	0
15. Repeat initial alert tests within 5 working days	1	0	0	0	0

S2 Table. Laboratory data (cont).

Data Characteristics	Concordance n/N				
	Cycle-1 (N= 70)	Cycle-2 (N=162)	Cycle-3 (N=85)	Cycle-4 (N=68)	Cycle-5 (N= 46)
1. EGASP number	70/70	162/162	85/85	67/68	46/46
2. Visit Date (DD/MM/YYYY)	70/70	161/162	84/85	68/68	46/46
3. Gram stain result from urethral swab (at clinic)	70/70	161/162	85/85	68/68	46/46
4. Morphologically typical colonies	35/70	162/162	85/85	68/68	45/46
5. Final Identification	70/70	162/162	85/85	68/68	43/46
6. Isolation storage date	70/70	157/162	82/85	68/68	46/46
7. NG Isolates available for AST after re-culture from freezing media (growth, no growth, contaminate)	36/70	162/162	84/85	52/68	46/46
8. CRO value	70/70	162/162	85/85	68/68	45/46
9. CFM value	70/70	162/162	85/85	68/68	46/46
10. AZI value	70/70	162/162	85/85	68/68	46/46
11. GEN value	70/70	162/162	85/85	68/68	45/46
12. CIP value	70/70	161/162	85/85	68/68	46/46
13. AST report date	70/70	162/162	85/85	68/68	46/46
14. Initial alert date/by	69/70	162/162	85/85	68/68	46/46
15. Repeat initial alert tests within 5 working days	69/70	162/162	85/85	68/68	46/46

S2 Table. Laboratory data (cont).

Data Characteristics	Concordance %					Cycle-1/2		Cycle-2/3		Cycle-3/4		Cycle-4/5	
	Cycle-1 (N= 70)	Cycle-2 (N=162)	Cycle-3 (N=85)	Cycle-4 (N=68)	Cycle-5 (N= 46)	OR (CI)	Fisher exact P-	OR (CI)	Fisher exact P-	OR (CI)	Fisher exact P-	OR (CI)	Fisher exact P-
1. EGASP number	100	100	100	98.5	100	1.006 (0.994 - 1.018)	1.000	0.994 (0.982 - 1.006)	1.000	1.015 (0.986 - 1.045)	0.444	0.985 (0.957 - 1.014)	1.000
2. Visit Date (DD/MM/YYYY)	100	99.4	98.8	100	100	1.006 (0.994 - 1.018)	1.000	1.006 (0.980 - 1.032)	1.000	0.988 (0.966 - 1.011)	1.000	N/A*	1.000
3. Gram stain result from urethral swab (at clinic)	100	99.4	100	100	100	1.006 (0.994 - 1.018)	1.000	0.994 (0.982 - 1.006)	1.000	N/A*	1.000	N/A*	1.000
4. Morphologically typical colonies	50	100	100	100	97.8	0.500 (0.396 - 0.632)	<0.001	N/A*	1.000	N/A*	1.000	1.022 (0.979 - 1.067)	0.404
5. Final Identification	100	100	100	100	87.4	N/A*	1.000	N/A*	1.000	N/A*	1.000	1.070 (0.991 - 1.155)	0.063
6. Isolation storage date	100	96.9	96.5	100	100	1.032 (1.004 - 1.061)	0.326	1.005 (0.956 - 1.005)	1.000	0.965 (0.926 - 1.005)	0.254	N/A*	1.000
7. NG Isolates available for AST after re-culture from freezing media (growth, no growth, contaminate)	51.4	100	98.8	76.5	100	0.514 (0.410 - 0.646)	<0.001	1.012 (0.989 - 1.036)	0.344	1.292 (1.130 - 1.477)	<0.001	0.765 (0.670 - 0.872)	<0.001
8. CRO value	100	100	100	100	97.8	N/A*	1.000	N/A*	1.000	N/A*	1.000	1.022 (0.979 - 1.067)	0.404
9. CFM value	100	100	100	100	100	N/A*	1.000	N/A*	1.000	N/A*	1.000	N/A*	1.000
10. AZI value	100	100	100	100	100	N/A*	1.000	N/A*	1.000	N/A*	1.000	N/A*	1.000
11. GEN value	100	100	100	100	97.8	N/A*	1.000	N/A*	1.000	N/A*	1.000	1.022 (0.979 - 1.067)	0.404
12. CIP value	100	99.4	100	100	100	1.006 (0.994 - 1.018)	1.000	0.994 (0.982 - 1.006)	1.000	N/A*	1.000	N/A*	1.000
13. AST report date	100	100	100	100	100	N/A*	1.000	N/A*	1.000	N/A*	1.000	N/A*	1.000
14. Initial alert date/by	98.6	100	100	100	100	0.986 (0.958 - 1.014)	0.302	N/A*	1.000	N/A*	1.000	N/A*	1.000
15. Repeat initial alert tests within 5 working days	98.6	100	100	100	100	0.986 (0.958 - 1.014)	0.302	N/A*	1.000	N/A*	1.000	N/A*	1.000

*No statistics are computed, variable is a constant.
