# Supplementary material for: Data quality assessment of the Enhanced Gonococcal Antimicrobial Surveillance Programme (EGASP), Thailand, 2015–2021
Source: PLoS One. 2024 Jul 5;19(7):e0305296. doi: 10.1371/journal.pone.0305296 (PMC11226028; doi:10.1371/journal.pone.0305296)
Supplement: S3 Table — (PDF) [file pone.0305296.s003.pdf]

S3 Table. Clinical data.

Data Characteristics	Discordance n/N				
	Cycle-1 (N= 70)	Cycle-2 * (N =157)	Cycle-3 (N=85)	Cycle-4 (N=68)	Cycle-5 **(N=42)
1. EGASP number	0	0	1	0	0
2. Visit Date (DD/MM/YYYY)	0	1	1	0	0
3. Gender of sex partner	7	8	1	1	2
4. Antibiotic use in the previous 2 weeks	0	5	2	1	2
5. Urethral gram stain result (only GNID)	2	1	0	0	0
6. Diagnosis at current visit	3	10	2	2	0
7. Primary treatment for gonorrhea	10	1	0	1	1
8. Presence of dual treatment for gonorrhea	10	3	0	2	1

S3 Table. Clinical data (cont).

Data Characteristics	Concordance n/N				
	Cycle-1 (N= 70)	Cycle-2 * (N =157)	Cycle-3 (N=85)	Cycle-4 (N=68)	Cycle-5 **(N=42)
1. EGASP number	70/70	157/157	84/85	68/68	42/42
2. Visit Date (DD/MM/YYYY)	70/70	156/157	84/85	68/68	42/42
3. Gender of sex partner	63/70	149/157	84/85	67/68	40/42
4. Antibiotic use in the previous 2 weeks	70/70	152/157	83/85	67/68	40/42
5. Urethral gram stain result (only GNID)	68/70	156/157	85/85	68/68	42/42
6. Diagnosis at current visit	67/70	147/157	83/85	66/68	42/42
7. Primary treatment for gonorrhea	60/70	156/157	85/85	67/68	41/42
8. Presence of dual treatment for gonorrhea	60/70	154/157	85/85	66/68	41/42

S3 Table. Clinical data (cont).

Data Characteristics	Concordance (%)					Cycle-1/2		Cycle-2/3		Cycle-3/4		Cycle-4/5	
	Cycle-1 (N= 70)	Cycle-2* (N =157)	Cycle-3 (N=85)	Cycle-4 (N=68)	Cycle-5 **(N=42)	OR (CI)	Fisher exact P-value	OR (CI)	Fisher exact P-value	OR (CI)	Fisher exact P-value	OR (CI)	Fisher exact P-value
1. EGASP number	100	100	98.8	100	100	N/A*	1.000	1.012 (0.989 - 1.036)	0.351	0.988 (0.966 - 1.011)	1.000	N/A*	1.000
2. Visit Date (DD/MM/YYYY)	100	99.4	98.8	100	100	1.006 (0.994 - 1.019)	1.000	1.005 (0.979 - 1.032)	1.000	0.988 (0.966 - 1.011)	1.000	N/A*	1.000
3. Gender of sex partner	90	94.9	98.8	98.5	95.2	0.948 (0.870 - 1.034)	0.245	0.960 (0.920 - 1.003)	0.166	1.003 (0.966 - 1.041)	1.000	1.035 (0.961 - 1.114)	0.557
4. Antibiotic use in the previous 2 weeks	100	96.8	97.6	98.5	95.2	1.033 (1.004 - 1.063)	0.327	0.991 (0.949 - 1.036)	1.000	0.991 (0.948 - 1.036)	1.000	1.035 (0.961 - 1.114)	0.557
5. Urethral gram stain result (only GNID)	97.1	99.4	100	100	100	0.978 (0.937 - 1.020)	0.226	0.994 (0.981 - 1.006)	1.000	N/A*	1.000	N/A*	1.000
6. Diagnosis at current visit	95.7	93.6	97.6	97.1	100	1.022 (0.959 - 1.090)	0.759	0.959 (0.910 - 1.011)	0.223	1.006 (0.954 - 1.061)	1.000	0.971 (0.931 - 1.012)	0.524
7. Primary treatment for gonorrhea	85.7	99.4	100	98.5	97.6	0.863 (0.783 - 0.950)	<0.001	0.994 (0.981 - 1.006)	1.000	1.015 (0.986 - 1.045)	0.444	1.009 (0.955 - 1.067)	1.000
8. Presence of dual treatment for gonorrhea	85.7	98.1	100	97.1	97.6	0.874 (0.792 - 0.964)	0.001	0.981 (0.960 - 1.003)	0.554	1.030 (0.989 - 1.074)	0.196	0.994 (0.934 - 1.059)	1.000

*No statistics are computed, variable is a constant.
