# Supplementary material for: Data quality assessment of the Enhanced Gonococcal Antimicrobial Surveillance Programme (EGASP), Thailand, 2015–2021
Source: PLoS One. 2024 Jul 5;19(7):e0305296. doi: 10.1371/journal.pone.0305296 (PMC11226028; doi:10.1371/journal.pone.0305296)
Supplement: S4 Table — (PDF) [file pone.0305296.s004.pdf]

S4 Table. Overall accuracy and completeness.

Attributes	Data parts	Document reviews (n/N, %)					% of improvement (5th-1st)	Fisher exact P-value *(cycle-1/cycle-2)*	Fisher exact P-value(cycle-2/cycle-3)	Fisher exact P-value (cycle-3/cycle-4)	Fisher exact P-value (cycle-4/cycle-5)
		1st	2nd	3rd	4th	5th					
**Accuracy**	**Clinical**	528/560, 94.3	1227/1256, 97.7	673/680, 98.9	537/544, 98.7	330/336, 98.2	3.9	<0.001	0.052	0.789	0.574
	**Laboratory**	979/1050, 93.2	2422/2429, 99.7	1270/1275, 99.6	1003/1020, 98.3	684/690, 99.1	5.9	<0.001	0.561	0.002	0.201
**Completeness**	**Clinical**	550/560, 98.2	1255/1256, 99.9	679/680, 99.9	542/544, 99.6	334/336, 99.4	1.2	<0.001	1.000	0.588	0.639
	**Laboratory**	980/1050, 93.3	2429/2430, 99.9	1273/1275, 99.8	1004/1020, 98.4	686/690, 99.4	6.1	<0.001	0.274	<0.001	0.069

Attributes	Document reviews (%, n/N)					*Improvement (cycle-1/cycle-5)*	Fisher exact P-value *(cycle-1 /cycle-2)*	Fisher exact P-value (cycle-2/cycle-3)	Fisher exact P-value (cycle-3/cycle-4)	Fisher exact P-value (cycle-4/cycle-5)
	**Cycle-1**	**Cycle-2**	**Cycle-3**	**Cycle-4**	**Cycle-5**					
**Overall accuracy**	93.6, 1507/1610	98.9, 3649/3686	99.4, 1943/1955	98.4, 1540/1564	98.8, 1014/1026	5.2	<0.001	0.174	0.010	0.496
**Overall completeness**	95.0, 1530/1610	99.9 3684/3686	99.8, 1952/1955	98.8, 1546/1564	99.4, 1020/1026	4.4	<0.001	0.349	<0.001	0.207
